# 1652. Impact of the 2022-2023 Amoxicillin Shortage on Antibiotic Prescribing for Acute Otitis Media: A Regression Discontinuity Study

**DOI:** 10.1093/ofid/ofad500.1486

**Published:** 2023-11-27

**Authors:** Rohan Khazanchi, Ryan Brewster, Alex Butler, Daniel O’Meara, Devika P Bagchi, Kenneth Michelson

**Affiliations:** Brigham and Women’s Hospital, Boston Children’s Hospital, and Boston Medical Center, Jamaica Plain, Massachusetts; Boston Children's Hospital, Boston, Massachusetts; Boston Children’s Hospital, Boston, Massachusetts; Boston Children's Hospital, Boston, Massachusetts; Boston Combined Residency Program at Boston Children’s Hospital and Boston Medical Center, Boston, Massachusetts; Boston Children's Hospital, Boston, Massachusetts

## Abstract

**Background:**

On October 28th, 2022, the U.S. Food and Drug Administration (FDA) announced a national shortage of amoxicillin powder for liquid suspension. The extent to which drug shortages affect prescribing patterns has been poorly described. To characterize the causal impact of the amoxicillin shortage, we performed a quasi-experimental analysis of antibiotic prescribing for uncomplicated acute otitis media (AOM).

**Methods:**

We retrospectively analyzed encounters for children < 18 years old diagnosed with AOM from May 15th, 2022 to April 14th, 2023 at an urban children's health system. Exclusion criteria included a prior episode of AOM within 30 days, presence of concomitant respiratory infections (sinusitis, pharyngitis, pneumonia, and conjunctivitis), and penicillin or amoxicillin allergy. We used a sharp regression discontinuity design with logistic regressions to evaluate the causal impact of the FDA shortage on the odds of being prescribed amoxicillin, amoxicillin-clavulanate, cefdinir, other antibiotics, or no antibiotic.

**Results:**

We identified 3076 encounters with 1677 (54.5%) occurring after the amoxicillin shortage (**Table 1**). The mean patient age was 4.1 years (SD 3.5), and most patients were male (n=1695, 55.1%), Hispanic (n=1252, 40.7%), and seen in the emergency department (n=1900, 61.8%). The most prescribed antibiotics were amoxicillin (n=1662, 54.0%), amoxicillin-clavulanate (n=671, 21.8%), and cefdinir (n=263, 8.6%).

The odds of being prescribed amoxicillin decreased by 91% after the FDA shortage declaration (OR 0.09 [95%CI 0.07-0.12]) (**Fig. 1A**). The odds of being prescribed amoxicillin-clavulanate or cefdinir increased 7-fold and 9-fold (7.90 [5.59-11.31] and 9.25 [5.27-17.17]), respectively (**Fig. 1B/1C**). Management without antibiotics (0.82 [0.55-1.23]) and prescriptions for other antibiotics (1.73 [0.96-3.19]) did not significantly change (**Fig. 1D/1E**).

**Table 1.**
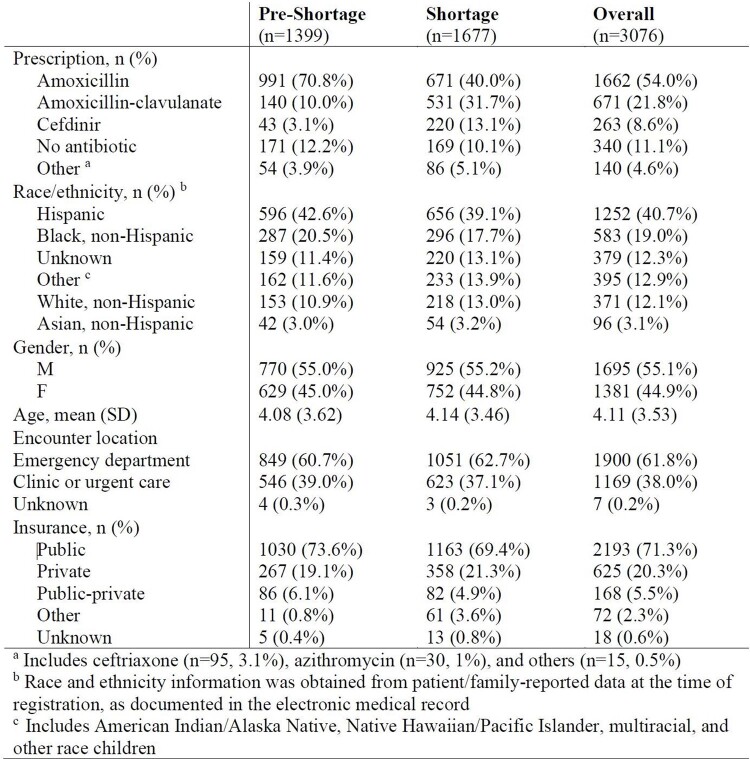
Encounter Characteristics for Patients Diagnosed with Acute Otitis Media During the Pre-Shortage (May 15, 2022 - October 27, 2022) and Shortage (October 28, 2022 - April 14, 2023) Periods

**Figure 1.**
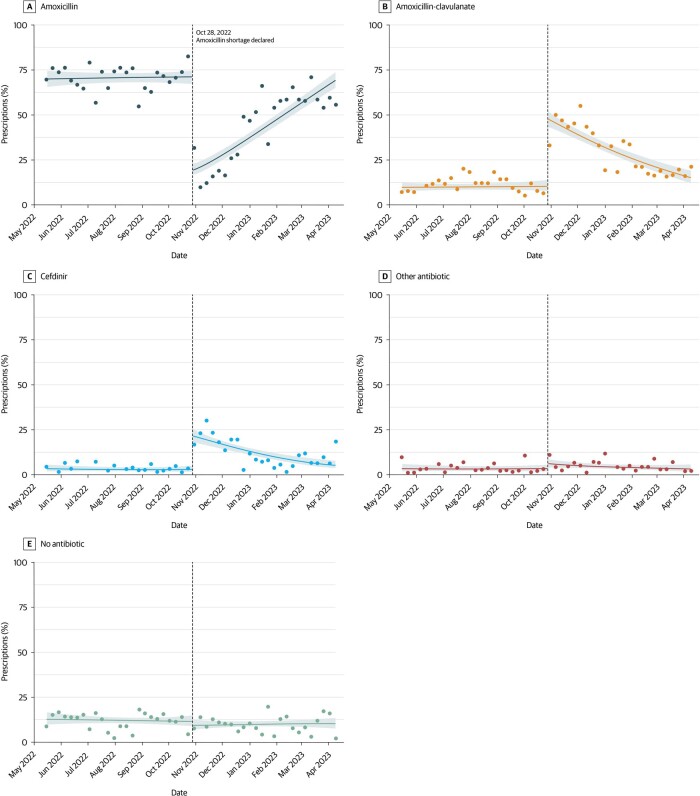
Regression Discontinuity Plot of Antibiotic Prescriptions Before and After the FDA Amoxicillin Shortage Declaration (October 28, 2022).

**Conclusion:**

The amoxicillin shortage had an immediate, sweeping impact on prescribing patterns. To mitigate future clinical, economic, and antimicrobial resistance consequences, the FDA should increase oversight of manufacturers responsible for critical medications, require supply issue resolution timelines, and incentivize antibiotic production to mitigate their low profitability.

**Disclosures:**

**Kenneth Michelson, MD**, Moderna: Stocks/Bonds|Pfizer: Stocks/Bonds

